# Prevalence of latent tuberculosis infection and feasibility of TB preventive therapy among Thai prisoners: a cross-sectional study

**DOI:** 10.1186/s12889-021-11271-0

**Published:** 2021-06-24

**Authors:** Sivaporn Gatechompol, Weerakit Harnpariphan, Ruamthip Supanan, Gompol Suwanpimolkul, Jiratchaya Sophonphan, Sasiwimol Ubolyam, Stephen J. Kerr, Anchalee Avihingsanon, Kamon Kawkitinarong

**Affiliations:** 1 HIV-NAT, Thai Red Cross AIDS Research Centre, 104 Ratchadamri Rd., Pathumwan, Bangkok, 10330 Thailand; 2 Tuberculosis Research Unit, Faculty of Medicine, Chulalongkorn University, Bangkok, Thailand; 3 Medical Correctional Institution, Bangkok and Klong Prem Central Prison, Bangkok, Thailand; 4 King Chulalongkorn Memorial Hospital, Thai Red Cross Society, Bangkok, Thailand; 5 Department of Medicine, Faculty of Medicine, Chulalongkorn University, Bangkok, Thailand; 6 Biostatistics Excellence Centre, Faculty of Medicine, Chulalongkorn University, Bangkok, Thailand

**Keywords:** LTBI, Inmate, Isoniazid preventive therapy, TST, IGRA

## Abstract

**Background:**

Prisons are considered as major reservoirs for tuberculosis. Preventive therapy for latent TB infection (LTBI) is an adjunctive strategy to control TB. However, LTBI data in Thai prisoners is limited. This study assessed the prevalence of LTBI and feasibility of isoniazid preventive therapy (IPT).

**Methods:**

A cross-sectional study was conducted among prisoners in Klong Prem Central Prison, Bangkok. Participants were screened for active TB by questionnaire and chest X-ray. LTBI was evaluated by Tuberculin skin test (TST) and QuantiFERON-TB Gold Plus (QFTP) among subgroup. Participants with positive TST or QFTP were considered to have LTBI. Participants with LTBI were offered IPT.

**Results:**

From August 2018–November 2019, 1002 participants were analyzed. All participants were male with a median age of 38 (IQR 32–50) years. LTBI identified by either TST/QFTP was present in 466 (46.5%) participants. TST was positive in 359 (36%) participants. In the subgroup of 294 participants who had both TST and QFTP results, 181/294 (61.6%) tested positive by QFTP. Agreement between TST and QFTP was 55.1% (Kappa = 0.17). The risk factors associated with LTBI were previous incarceration (aOR 1.53, 95%CI, 1.16–2.01, *p* = 0.002), history of prior active TB (aOR 3.02, 95%CI, 1.74–5.24, *p* < 0.001) and duration of incarceration ≥10 years (aOR 1.86, 95%CI, 1.24–2.79, *p* = 0.003). Majority of LTBI participants (82%) agreed to take IPT. Three hundred and 56 (93%) participants completed treatment whereas 27 (7%) participants discontinued IPT due to the side effects of INH.

**Conclusion:**

This is the first study to evaluate the prevalence of LTBI and feasibility of IPT among Thai prisoners. LTBI prevalence in male prisoners in Thailand is high. LTBI screening and treatment should be implemented together with other preventive components.

## Background

In 2019, 10 million people developed tuberculosis (TB) and approximately 1.4 million people died from TB-related illnesses worldwide [1]. Although cumulative TB incidence reduced approximately 9% from 2015 to 2019, this less than half of the 20% reduction required to meet the World Health Organization (WHO)‘s End TB Strategy milestone between 2015 and 2020 [2]. One challenge is the high TB incidence among high risk populations, including prisoners. Globally, TB incidence is 5 to 70 times greater in prisons than in general population [3].

Prisons act as an institutional TB amplifier, facilitating transmission to the general population through released inmates, especially in low-middle income countries [4]. High levels of incarceration therefore contribute to the increasing TB incidence in the general population level [5]. Many prisoners are from populations at high risk for TB infection and active TB disease, such as alcohol or drug users and people living with human immunodeficiency virus (HIV) [6]. Structural and institutional factors contributing to transmission of TB in prisons include poor ventilation, overcrowding, delayed diagnosis and inadequate treatment.

Thailand is one of 14 countries with the highest burden of TB, TB/HIV and multi drug resistance (MDR)-TB defined by WHO [7]. Moreover, Thailand had the 6th highest number of incarcerated individuals worldwide in 2021 [8]. A nationwide active TB screening campaign was conducted in 2017. Using chest X-ray (CXR) in addition to symptom screening among 285,367 inmates in 143 prisons, a total of 2473 active tuberculosis cases (873/100,000 persons) were detected [9], a rate 5.6 times higher than in the general population [1]. Although active case finding and treatment of active TB remain the keys for TB control, this approach alone is not sufficient to achieve the WHO End TB Strategy targets: TB preventive therapy for latent TB infection (LTBI) is a necessary adjunctive strategy [10].

There are currently no data that describe LTBI rates in Thai prisoners. Our primary objective was therefore to assess the prevalence of LTBI based on tuberculin skin test (TST) and/or interferon gamma release assay (IGRA), and assess agreement between tests among Thai prisoners tested with both. We also evaluated the feasibility and uptake of latent tuberculosis preventive therapy in this population.

## Methods

### Study design and participants

From August 2018 to November 2019, we conducted a cross-sectional study in Klong Prem Central Prison, a maximum-security prison in Bangkok, Thailand. In 2018, the incidence of TB in this prison was 1077 cases per 100,000 persons. It currently houses more than 6000 inmates, all of whom are male. The inclusion criteria for this study were Thai inmates, aged ≥18 years, with more than 2 years remaining in their prison terms to enable follow-up of TB incidence. Participants diagnosed with active TB within the previous 6 months were excluded.

After TB education sessions and information describing the study were delivered by study site staffs, interested participants were asked to provide written informed consent. The consent process and all study procedures were conducted in Thai, by study site staffs.

### Ethical approval

This study was approved by the Department of Corrections, Ministry of Justice, Thailand and the ethics committees of Chulalongkorn University. This study was conducted according to Declaration of Helsinki and Good Clinical Practice. All participants provided signed written informed consent.

### Study procedures

We collected participant information by using a questionnaire. The information included demographic data, history of incarceration, HIV risk behaviors, history of substance misuse, history of close contact TB, history of previous TB diseases and current TB symptoms. Participants underwent portable chest radiographs, which were read by radiologists. Those testing positive on symptom screening or chest radiographs were investigated further using sputum smear microscopy, culture for *M. tuberculosis* and/or GeneXpert MTB/ rifampin (RIF) (Xpert; Cepheid, Sunnyvale, CA). Participants diagnosed with active TB were sent to the Medical Correctional hospital located in the same area as the Prison, for TB treatment and isolation. Participants also underwent blood testing for HIV antibody, hepatitis B surface antigen (HBs Ag), Anti HCV and syphilis (by venereal disease research laboratory with a confirmatory test).

### LTBI testing and interpretation

For all participants, the one step TST protocol was administered using two tuberculin units (0.1 mL) of RT23 Purified Protein Derivative (PPD; Beijing Sanroad biological products co., Ltd) on the left forearm using the intradermal technique. The size of induration was measured by trained study nurses after 48–72 h using the ballpoint reader method. An induration of ≥10 mm, or in the case of an HIV positive participant ≥5 mm, was considered positive.

Due to the high expense involved with IGRA and limited study budget, only the first 294 participants that were enrolled into the study had IGRA testing in addition to TST. Blood samples for QuantiFERON-TB Gold Plus (QFTP, Qiagen, Valencia, CA, USA) testing were collected by venipuncture prior to TST and performed according to the manufacturers’ instructions. QFTP includes two antigen tubes, TB1 and TB2 which contain specific *Mycobacterium tuberculosis* (MTB) peptides. TB1 and TB2 are designed to target cell mediated responses from cluster of differentiation (CD) 4 T-cells and CD8 T-cells, respectively [11].

TST and QFTP readings were performed independently; TST by study nurses as described above. QFTP was performed and analyzed by medical technologists. Nurses and medical technologists were blinded from the result of the corresponding test. Participants with positive TST or QFTP were considered as having LTBI. According to National Tuberculosis Control Programme Guideline of Thailand, LTBI treatment is not recommended for prisoner [12]. However, participants with LTBI in this study were offered daily isoniazid preventive therapy (IPT) for 6 months or 9 months for HIV negative and HIV positive participants, respectively. Participants who received IPT were followed up at 1 month, and every 3 months thereafter if there was no evidence of adverse events. Participants received IPT under direct observed therapy (DOT) by prison healthcare staff. IPT completion was defined as taking IPT for at least 90% of the total designated dose for 6 months or 9 months according to their HIV status.

### Statistical analysis

Baseline characteristics of participants were stratified by LTBI status. Formal comparisons between the LTBI group and non- LTBI group were made using a chi-square or Wilcoxon rank-sum test for categorical and continuous variables, respectively. Multivariable logistic regression was used to determine factors associated with LTBI prevalence diagnosed by TST or QFTP, factors associated with QTFP+/TST- discordant results (versus positive concordant results) and development of adverse events necessitating treatment cessation in those patients who started IPT. We assessed the linearity of continuous predictors against the logit function, and in the case of non-linearity, the variable was modelled in quartiles. Adjacent quartiles were collapsed together if the odds ratio and 95%CI were similar. Variables with *p* < 0.1 in univariate models were adjusted for in multivariable models.

A subgroup analysis of participants who had both TST and QFTP testing were performed to assess the consistency of the two tests for diagnosing LTBI. The agreement between the TST and QFTP was determined using Cohen’s kappa statistic (κ), with coefficients of > 0.75, 0.45–0.75 and < 0.4 considered as excellent, fair to good and poor agreement, respectively. A sensitivity analysis compared agreement between IGRA and TST in patients with TST induration > 5 mm given that prisoners live in very crowded conditions and are at high risk for contracting active TB. McNemar’s test of paired proportions was used to generate a *P*-value for the concordance of the tests. All statistical tests were two-sided, at a nominal 5% significance level. Statistical analyses were performed using Stata 15.1 (StataCorp, College Station, TX, USA).

## Results

### Participant characteristics

Among the total of 6194 inmates in this prison, 3700 inmates who have more than 50 years sentence were located in the highest security zone. We were not authorized to conduct research in this area. One thousand twenty inmates were excluded as the duration of their remaining prison terms was < 2 years, and early release would preclude collection of follow-up data. Hence, 1474 inmates were met the inclusion criteria. Of these, 1032 (70%) inmates were interested in participating in this study. Thirty participants were excluded because of abnormal chest X-rays (*n* = 25) and incomplete TST testing (*n* = 5). Thus, 1002 participants were included in the analysis. All participants were male with median age of 38 (interquartile range (IQR) 32–50) years. Median duration of current incarceration was 5.8 (IQR 3.8–8.2) years. The prevalence of HIV, hepatitis B virus (HBV), hepatitis C virus (HCV) and syphilis infections were 2.9, 6.1%, 5.7 and 4.9%, respectively. Participants’ characteristics stratified by LTBI status are shown in Table 1.

**Table 1:** Participant characteristics stratified by LTBI status

	Total *N* = 1002	No LTBI *N* = 536	LTBI *N* = 466	P
Age (years), median (IQR)	38 (32–50)	38 (31–49)	38 (32–52)	0.07
Body mass index (kg/m^2^), median (IQR)	21.7 (19.8–23.5)	21.8 (20–23.7)	21.6 (19.6–23.5)	0.11
Highest education, n (%)				0.53
• Elementary	621 (62)	324 (60.5)	297 (63.7)	
• High school	299 (29.8)	164 (30.5)	135 (29.0)	
• Bachelor or higher	82 (8.2)	48 (9)	34 (7.3)	
Duration in prison (years), median (IQR)	5.8 (3.8–8.2)	5.6 (3.4–7.8)	6.3 (4.2–9.1)	0.001
History of previous incarceration, n (%)	307 (30.8)	140 (26.3)	167 (36.1)	0.001
History of substance abuse, n (%)	626 (62.5)	323 (60.3)	303 (65)	0.12
History of previous active TB, n(%)	76 (7.6)	21 (3.9)	55 (11.8)	< 0.001
HIV seropositive, n(%)	29 (2.9)	10(1.9)	19(4)	0.04

### Prevalence of LTBI and factors associated with LTBI in prison

LTBI identified by either TST or QFTP positivity was present in 466/1002 (46.5%) participants. In univariable logistic regression of factors associated with LTBI, age ≥ 30 years, having been incarceration ≥10 years, a history of previous incarceration, a history of active TB and HIV infection had *p*-values < 0.1, and were entered into the multivariate model. In the multivariable model, having been currently incarcerated ≥10 years (adjusted odds ratio (aOR) 1.86 [95% confidence interval (CI), 1.24–2.79] *p* = 0.003), a history of previous incarceration (aOR 1.61 [95%CI, 1.22–2.13] *p* = 0.001) and history of active TB (aOR 2.77 [95%CI, 1.59–4.82] *p* < 0.001) remained independently associated with LTBI prevalence (Table 2).

### Test agreement between TST and QFT-plus

Overall TST was positive in 359/1002 (35.8%) participants. In the subgroup of 294 participants who had both TST and QFTP results available, 99/294 (33.7%) tested positive by TST and 181/294 (61.6%) tested positive by QFTP. Of these, 74 (25.2%) had concordant positive results, 88 (29.9%) had concordant negative results and 107 (36.4%) had QFTP +/TST- discordance. Only 25(8.5%) had QFTP−/TST+ discordance.

In univariable logistic regression analysis, history of previous active TB and history of contact with a known TB case had *p* < 0.1, and were entered into the in the multivariable model. In this model, only history of previous active TB remained independently associated with QTFP+/TST- discordance (aOR 0.26, 95% CI 0.10, 0.6, *p* = 0.005) after adjusting for known contact with a TB case, suggesting those with known previous active TB were less likely to have a QTFP+/TST- discordant versus positive concordant response. We compared age and weight between QTFP+/TST- and QTFP−/TST+ discordance groups, but the distribution of the variables between the groups was similar.

Agreement between tests was 55.1% (Kappa = 0.17 (95% CI 0.071–0.259) (Table 3).

**Table 2:** Factor associated with LTBI in prison

	Univariate		Multivariate	
	OR (95%CI)	*P*-value	aOR (95%CI)	*P*-value
Age ≥ 30 years	1.36 (0.97–1.91)	0.07	1.13 (0.79–1.62)	0.49
Body mass index < 18.5 kg/m^2^	1.38 (0.93–2.03)	0.11		
Duration in prison (years)				
< 5 years	Ref		Ref	
5–9	1.23 (0.94–1.62)	0.13	1.18 (0.89–1.58)	0.25
≥ 10	1.90 (1.30–2.79)	0.001	1.86 (1.24–2.79)	0.003
History of previous incarceration	1.58 (1.21–2.07)	0.001	1.61(1.22–2.13)	0.001
History of active TB	3.28 (1.95–5.52)	< 0.001	2.77 (1.59–4.82)	< 0.001
History of substance abuse	1.23 (0.95–1.59)	0.12		
History of contact TB cases	1.23 (0.86–1.76)	0.26		
HIV co-infection	2.24 (1.03–4.89)	0.04	1.32 (0.55–3.14)	0.54
HBV co-infection	1.20 (0.72–2.02)	0.49		
HCV co-infection	1.39 (0.82–2.39)	0.22

**Table 3:** Paired Results of testing with TST and QFTP

TST	QFTP		Total
	Positive	Negative	
Positive	74	25	99
Negative	107	88	195
Total	181	113	294

TST positivity rate: 99/294 = 33.7%

QFTP positivity rate: 181/294 = 61.6%

Kappa score (95%CI): 0.165 (0.071–0.259)

*QFTP* QuantiFERON-TB Gold Plus, *TST* Tuberculin skin test

Agreement was also low at 60.2% when the cut-off for a positive TST was reduced to > 5 mm for all patients (Kappa = 0.11 (95%CI 0.001–0.226); McNemar’s P for both comparisons < 0.001.

**Fig. 1:**
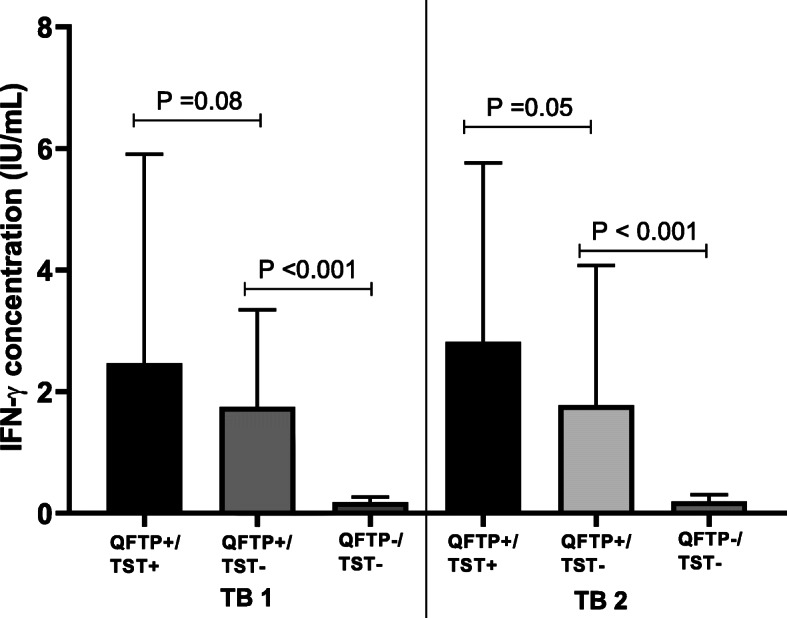
Participants with discordant QFTP+/TST- had lower IFN- γ production as compared with concordant positive QFTP+/TST+. QFTP; QuantiFERON-TB Gold Plus, TST; Tuberculin skin test

In the assessment where a positive TST result was judged as > 10 mm for participants except those with HIV-infection, participants in QFTP +/TST- discordance group had lower median IFN-γ concentrations in both TB1 and TB2 tubes than participants who had concordant positive (QFTP +/TST+) results (TB1: 1.75 IU/mL (IQR 0.89–3.34) vs 2.47 IU/mL (IQR 0.96–5.84); *p* = 0.08, TB2: 1.78 IU/mL (IQR 1.04–4.03) vs 2.82 IU/mL (1.41–5.69); *p* = 0.05) Fig. 1.

### LTBI treatment initiation and completion

The majority of LTBI participants (*n* = 383; 82.2%) agreed to start IPT. Of these, 356/383 (92.9%) completed treatment and 27 (7.1%) participants discontinued IPT due to adverse effects. The most common side effects were nausea (2.6%), vomiting (2.3%) and rash (2.1%). Two participants (0.5%) developed drug-induced hepatitis necessitating IPT discontinuation. Having HBV co-infection (aOR 4.92, 95%CI, 1.76–13.78, *p* = 0.002) and HCV co-infection (aOR 3.56, 95%CI, 1.12–10.59, *p* = 0.02) were associated with gastrointestinal (GI) adverse events and hepatotoxicity after IPT initiation.

## Discussion

This study evaluated prevalence of LTBI, by using TST and QFTP, in a large prison in Bangkok, Thailand, and to our knowledge, is the first study to evaluate LTBI prevalence and feasibility of LTBI treatment among Thai prisoners. Surprisingly the 61.6% prevalence of positive QFTP was much higher than the 13 and 20% prevalence in reported in Thai people living with HIV (PLWH) [13] and Thai health care workers, respectively [14] who were also tested by IGRA. Furthermore, this positive IGRA prevalence was higher than that reported from correctional facilities in other high TB burden countries such as China (52%) [15] and Ethiopia (51%) [16].

For TST, 35.8% of all participants, and 33.7% in the subgroup who additionally had IGRA testing, respectively, had a positive TST. These findings are similar to the prevalence of TST among Brazilian prisoners (33%) [17]. Whereas those reported from prisoners in other high TB burden countries ranged from 62 to 89% [18–20]. However, the prevalence of LTBI defined by TST in our study, was higher than that reported in Thai PLWH [13, 21–23].

Our study demonstrated poor agreement between TST and QFTP test results. We showed 36.4% prevalence of QFTP +/TST- discordance among prisoners in a TB high endemic country. These findings are in line with previous studies among other high-risk populations, including prisoners [24], health care workers [14, 25] and HIV infected individuals [26–28]. The immunologic explanation for this discordance, however, is poorly understood. The QFTP +/TST- discordance group tended to have lower IFN-γ production from CD4 T-cells and CD8 T-cells in response to MTB antigens than those with concordant positive results. The tuberculin skin reaction, a localized immune reaction to soluble MTB antigens, is a delayed type hypersensitivity reaction (DTH). Mycobacterial antigens drive the differentiation of CD4+ T cells to Th1 cells. Th1 cells secrete IFN- γ that is responsible for macrophage activation. Moreover, the effector mechanisms of DTH include coagulation and microvascular homeostasis, in addition to macrophage activation which all affect skin induration results [29, 30]. In contrast, IGRA only measures IFN-γ released from T-cell in the peripheral blood. Our findings suggest that lower IFN- γ concentrations may be below the threshold necessary to trigger DTH, resulting in a false negative TST. This is consistent with a previous study where lower IFN- γ and IL-2 levels were associated with IGRA+/TST- discordance in pregnant HIV-infected women [26]. A meta-analysis comparing the sensitivity of TST with QuantiFERON Gold In-tube (QFT-GIT) in active TB disease found a higher sensitivity of QFT-GIT compared to TST, suggesting that the low TST positive rate could be due in part to false negative results [31]. Another reason that explains this discordance may relate to operator dependent errors when performing and reading TST. However, we believe this explanation is less likely a contributor to the discordance, because both nurses were well trained and experienced in performing TST.

Having been incarcerated ≥10 years, or having a previous incarceration were independent risk factors associated with LTBI prevalence in our study. Many previous studies have demonstrated that previous incarceration was a risk factor for LTBI in prisons, regardless of the TB burden in the country [15, 17, 20, 32]. Furthermore, a longer duration of incarceration was associated with LTBI among Chinese [15] and Ethiopian [16] prisoners. This finding underscores the cumulative nature of the risk of TB transmission among prisoners, and highlights the public health importance of implementing LTBI screening and treatment in this setting.

Another key finding of our study is the feasibility of providing IPT in Thai prisoners. Ninety-three percent of participants who started IPT successfully completed their course. This rate is higher than the range of 3 to 87% reported in a systematic review among prisoners in international setting [33]. However, the majority of studies in this review were conducted in low TB endemic, high-income countries. We reported the high rate of IPT completion in response to WHO recommendation to focus on increased detection and early diagnosis in low- and middle-income prison settings [34]. We found 7% of participants discontinued IPT due to the side effects. Having HBV or HCV co-infection were significant risk factors for developing GI adverse event and hepatoxicity after IPT initiation. Hence, the testing for viral hepatitis should be consider before IPT initiation.

There are some limitations to our study. First, this is a cross-sectional study, which precludes analysis of longitudinal changes including incident TB among participants. Second, IGRA testing was not available in every participant due to lack of funding, which limits the power to fully evaluate the agreement between TST and IGRA. Third, all participants were males, so our results might not be applicable to female prisoners. Fourth, we did not assess the history of BCG vaccination among the participants, which may affect TST positivity. However, since 1967, Thailand has routinely vaccinated newborn babies on their first day of life. Therefore, based on participant age, 786 (78%) participants are likely to have been BCG vaccinated as babies, however BCG re-vaccination is not recommended in Thailand. Fifth, 442 of 1474 eligible inmates did not consent to participating in this project, and the possibility of some selection bias cannot be ruled out. Lastly, although the nurses conducting and interpreting the TST results were experienced in this technique, and our laboratory is accredited by the College of American Pathologists, there is a possibility that discordance may have arisen due to TST interpretation or experience with conducting the IGRA test. The discordance rate amongst the first and second half of the IGRA tests conducted in chronological order in the inmates who dual tested was similar at 39 versus 32%. In future studies, we would have more confidence in these discordant results if the TST results were independently checked by two nurses, and the QFT was confirmed by an independent laboratory.

LTBI prevalence in male prisoners in Thailand is high. Given that there is no gold standard to diagnose the true prevalence of LTBI, our study suggests that the QFTP may be superior to TST in identifying prisoners that would benefit from IPT in a TB high burden country. We also demonstrated high completion rates of IPT. In order to effectively control TB infection in correctional facilities, programs including LTBI screening and treatment should be implemented together with other preventive components such as active TB case-finding, infection control and early treatment of active TB.

## Data Availability

The datasets used and/or analyzed during the current study are available from the corresponding author and can access on reasonable request.

## Abbreviations

aOR: adjusted odds ratio
CD4: Cluster of differentiation 4
CD8: Cluster of differentiation 8
CI: Confidence interval
CXR: Chest X-ray
DTH: Delayed type hypersensitivity reaction
DOT: Direct observed therapy
GI: Gastrointestinal
HBs Ag: Hepatitis B surface antigen
HBV: Hepatitis B virus
HCV: Hepatitis C virus
HIV: Human immunodeficiency virus
IFN-γ: Interferon gamma
IGRA: Interferon gamma release assay
IQR: *Interquartile range*
IPT: Isoniazid preventive therapy
RIF: Rifampin
QFTP: QuantiFERON-TB Gold Plus
LTBI: Latent TB infection
MDR: Multi drug resistance
MTB: *Mycobacterium tuberculosis*
PLWH: People living with HIV
TST: Tuberculin skin test
TB: Tuberculosis
WHO: World Health Organization

## References

[1] World Health Organization. Global tuberculosis report 1997–2020. Geneva; 2020. https://www.who.int/teams/global-tuberculosis-programme/data. Accessed 20 Apr 2021

[2] World Health Organization. Tuberculosis. Key facts. Geneva; 2020. https://www.who.int/news-room/fact-sheets/detail/tuberculosis. Accessed 20 Apr 2021

[3] USAID. Tuberculosis in prisons: a growing public health challenge. USA; 2014. https://www.usaid.gov/sites/default/files/documents/1864/USAID-TB-Brochure.pdf. Accessed 20 Apr 2021

[4] Mathema B, Andrews JR, Cohen T, Borgdorff MW, Behr M, Glynn JR, Rustomjee R, Silk BJ, Wood R (2017). Drivers of tuberculosis transmission. J Infect Dis.

[5] Stuckler D, Basu S, McKee M, King L (2008). Mass incarceration can explain population increases in TB and multidrug-resistant TB in European and central Asian countries. Proc Natl Acad Sci U S A.

[6] World Health Organization. Tuberculosis in prisons. Geneva; 2013. https://www.who.int/tb/areas-of-work/population-groups/prisons/en/. Accessed 20 Apr 2021

[7] World Health Organization. Use of high-burden country lists for TB by WHO in the post-2015 era. Geneva; 2015. https://www.who.int/tb/publications/global_report/high_tb_burdencountrylists2016-2020.pdf. Accessed 20 Apr 2021

[8] World Prison Brief. Highest to Lowest - Prison Population Total. UK; 2020. https://www.prisonstudies.org/highest-to-lowest/prison-population-total?field_region_taxonomy_tid=All. Accessed 20 Apr 2021

[9] Jittimanee S, Vorasingha J, Poopatana S, Smithikarn S, Kamolwat P. Nationwide chest X-ray screening for active TB in Thai prisons [abstract]. The Hague: The 49th World Conference on Lung Health; 24-27 October 2018; Abstract no: PS 49-934-27

[10] Uplekar M, Weil D, Lonnroth K, Jaramillo E, Lienhardt C, Dias HM, Falzon D, Floyd K, Gargioni G, Getahun H, Gilpin C, Glaziou P, Grzemska M, Mirzayev F, Nakatani H, Raviglione M (2015). WHO’s new end TB strategy. Lancet.

[11] Allen NP, Swarbrick G, Cansler M, Null M, Salim H, Miyamasu M (2018). Characterization of specific CD4 and CD8 T-cell responses in QuantiFERON TB gold-plus TB1 and TB2 tubes. Tuberculosis.

[12] National Library of Thailand Cataloging in Publication Data. National Tuberculosis Control Programme Guideline. Thailand; 2018. https://www.tbthailand.org/download/Manual/NTP2018.pdf. Accessed 20 Apr 2021

[13] Khawcharoenporn T, Apisarnthanarak A, Phetsuksiri B, Rudeeaneksin J, Srisungngam S, Mundy LM (2015). Tuberculin skin test and QuantiFERON-TB gold in-tube test for latent tuberculosis in Thai HIV-infected adults. Respirology.

[14] Khawcharoenporn T, Apisarnthanarak A, Sangkitporn S, Rudeeaneksin J, Srisungngam S, Bunchoo S, Phetsuksiri B (2016). Tuberculin skin test and QuantiFERON((R))-TB gold in-tube test for diagnosing latent tuberculosis infection among Thai healthcare workers. Jpn J Infect Dis.

[15] Zhang G, Zhang Y, Zhong D, Meng S, An L, Wei W, Zhang Z, Fu Y, Wang X (2020). High prevalence of and risk factors for latent tuberculosis infection among prisoners, Tianjin, China. Emerg Infect Dis.

[16] Chekesa B, Gumi B, Chanyalew M, Zewude A, Ameni G (2020). Prevalence of latent tuberculosis infection and associated risk factors in prison in east Wollega zone of western Ethiopia. PLoS One.

[17] Carbone Ada S, Paiao DS, Sgarbi RV, Lemos EF, Cazanti RF, Ota MM (2015). Active and latent tuberculosis in Brazilian correctional facilities: a cross-sectional study. BMC Infect Dis.

[18] Mamani M, Mahmudian H, Majzoobi MM, Poorolajal J (2016). Prevalence and incidence rates of latent tuberculous infection in a large prison in Iran. Int J Tuberc Lung Dis.

[19] Guerra J, Mogollon D, Gonzalez D, Sanchez R, Rueda ZV, Parra-Lopez CA (2019). Active and latent tuberculosis among inmates in La Esperanza prison in Guaduas, Columbia. PLoS One.

[20] Al-Darraji HA, Kamarulzaman A, Altice FL (2014). Latent tuberculosis infection in a Malaysian prison: implications for a comprehensive integrated control program in prisons. BMC Public Health.

[21] Danyuttapolchai J, Kittimunkong S, Nateniyom S, Painujit S, Klinbuayaem V, Maipanich N, Maokamnerd Y, Pevzner E, Whitehead S, Kanphukiew A, Monkongdee P, Martin M (2017). Implementing an isoniazid preventive therapy program for people living with HIV in Thailand. PLoS One.

[22] Miyahara R, Piyaworawong S, Prachamat P, Wongyai J, Bupachat S, Yamada N, Summanapan S, Yanai H, Mahasirimongkol S (2020). High tuberculosis burden among HIV-infected populations in Thailand due to a low-sensitivity tuberculin skin test. J Infect Public Health.

[23] Phanuphak N, Varma JK, Kittikraisak W, Teeratakulpisarn N, Phasitlimakul S, Suwanmala P, Pankam T, Burapat T, Tasaneeyapan T, McCarthy KD, Cain KP, Phanuphak P (2012). Using tuberculin skin test as an entry point to screen for latent and active tuberculosis in Thai people living with HIV. J Acquir Immune Defic Syndr.

[24] Nijhawan AE, Iroh PA, Brown LS, Winetsky D, Porsa E (2016). Cost analysis of tuberculin skin test and the QuantiFERON-TB gold in-tube test for tuberculosis screening in a correctional setting in Dallas, Texas, USA. BMC Infect Dis.

[25] Adams S, Ehrlich R, Baatjies R, Dendukuri N, Wang Z, Dheda K (2019). Predictors of discordant latent tuberculosis infection test results amongst south African health care workers. BMC Infect Dis.

[26] Mathad JS, Bhosale R, Balasubramanian U, Kanade S, Mave V, Suryavanshi N, Gupte N, Joshi S, Chandanwale A, Dupnik KM, Kulkarni V, Deshpande P, Fitzgerald DW, Gupta A (2016). Quantitative IFN-gamma and IL-2 response associated with latent tuberculosis test discordance in HIV-infected pregnant women. Am J Respir Crit Care Med.

[27] Leidl L, Mayanja-Kizza H, Sotgiu G, Baseke J, Ernst M, Hirsch C, Goletti D, Toossi Z, Lange C (2010). Relationship of immunodiagnostic assays for tuberculosis and numbers of circulating CD4+ T-cells in HIV infection. Eur Respir J.

[28] Mandalakas AM, Hesseling AC, Chegou NN, Kirchner HL, Zhu X, Marais BJ, Black GF, Beyers N, Walzl G (2008). High level of discordant IGRA results in HIV-infected adults and children. Int J Tuberc Lung Dis.

[29] Hunter RL. The pathogenesis of tuberculosis-the Koch phenomenon reinstated. Pathogens. 2020;9(10). 10.3390/pathogens9100813. 10.3390/pathogens9100813 PMC7601602 33020397

[30] Elliott JH, Vohith K, Saramony S, Savuth C, Dara C, Sarim C, Huffam S, Oelrichs R, Sophea P, Saphonn V, Kaldor J, Cooper DA, Chhi Vun M, French MA (2009). Immunopathogenesis and diagnosis of tuberculosis and tuberculosis-associated immune reconstitution inflammatory syndrome during early antiretroviral therapy. J Infect Dis.

[31] Sester M, Sotgiu G, Lange C, Giehl C, Girardi E, Migliori GB, Bossink A, Dheda K, Diel R, Dominguez J, Lipman M, Nemeth J, Ravn P, Winkler S, Huitric E, Sandgren A, Manissero D (2011). Interferon-gamma release assays for the diagnosis of active tuberculosis: a systematic review and meta-analysis. Eur Respir J.

[32] Rueda ZV, Arroyave L, Marin D, Lopez L, Keynan Y, Giraldo MR (2014). High prevalence and risk factors associated with latent tuberculous infection in two Colombian prisons. Int J Tuberc Lung Dis.

[33] Al-Darraji HA, Kamarulzaman A, Altice FL (2012). Isoniazid preventive therapy in correctional facilities: a systematic review. Int J Tuberc Lung Dis.

[34] World Health Organization (2000). Tuberculosis Control in Prisons: a manual for programme managers.

